# The Cation/Calcium Channel of Sperm (CatSper): A Common Role Played Despite Inter-Species Variation?

**DOI:** 10.3390/ijms241813750

**Published:** 2023-09-06

**Authors:** Alejandro Vicente-Carrillo, Manuel Álvarez-Rodríguez, Heriberto Rodriguez-Martinez

**Affiliations:** 1 Department of Animal Production, Veterinary Faculty, Complutense University of Madrid, 28040 Madrid, Spain; 2 Department Animal Reproduction, Instituto Nacional de Investigación y Tecnología Agraria y Alimentaria-Consejo Superior de Investigaciones Científicas (INIA-CSIC), 28040 Madrid, Spain; 3 Department of Biomedical and Clinical Sciences (BKV), Linköping University, 58225 Linköping, Sweden

**Keywords:** calcium, CatSper, sperm cells, biomarker, sub-fertility, livestock

## Abstract

The main cation/calcium channel of spermatozoa (CatSper), first identified in 2001, has been thoroughly studied to elucidate its composition and function, while its distribution among species and sperm sources is yet incomplete. CatSper is composed of several subunits that build a pore-forming calcium channel, mainly activated in vivo in ejaculated sperm cells by intracellular alkalinization and progesterone, as suggested by the in vitro examinations. The CatSper channel relevance is dual: to maintain sperm homeostasis (alongside the plethora of membrane channels present) as well as being involved in pre-fertilization events, such as sperm capacitation, hyperactivation of sperm motility and the acrosome reaction, with remarkable species differences. Interestingly, the observed variations in CatSper localization in the plasma membrane seem to depend on the source of the sperm cells explored (i.e., epididymal or ejaculated, immature or mature, processed or not), the method used for examination and, particularly, on the specificity of the antibodies employed. In addition, despite multiple findings showing the relevance of CatSper in fertilization, few studies have studied CatSper as a biomarker to fine-tune diagnosis of sub-fertility in livestock or even consider its potential to control fertilization in plague animals, a more ethically defensible strategy than implicating CatSper to pharmacologically modify male-related fertility control in humans, pets or wild animals. This review describes inter- and intra-species differences in the localization, structure and function of the CatSper channel, calling for caution when considering its potential manipulation for fertility control or improvement.

## 1. Introduction

Mammalian sperm cells follow a process of biochemical and physiological sperm maturation alongside the epididymal duct, acquiring progressive motility and the ability to fertilize [1]. Sperm cells are thereafter stored, quiescent, in the epididymal cauda until emitted at ejaculation, where they eliminate the migrating remnant of cytoplasm—the distal cytoplasmic drop [1]. During this epididymal pre-ejaculatory period, calcium is mainly progressively accumulated in sperm intracellular depots, a source of calcium that is later required for sperm hyperactivation [2]. After mating/natural intercourse, ejaculated sperm cells become highly interactive cells, fully displaying their innate motility and biochemical interchangeability with the seminal plasma and the epithelium lining of the female genital tract [3]. These interactions are essential for the prerequisite of sperm capacitation that enables cohorts of sperm cells to be fit for fertilization of the oocyte, incorporating hyperactivation of their motility and the reactivity towards hyaluronan between the expanded cumulus cloud and the zona pellucida (ZP) proteins which, ending in the acrosome reaction, facilitates adjournment to the oolemma [4,5]. Basic to these processes is the movement of calcium ions from both the intracellular sperm deposits as well as from extracellular sources, incoming through the sperm plasma membrane [6,7]. The intracellular increase in calcium from the media is mediated by several calcium channels [8], among which the most relevant is the CatSper [9]. Noteworthy, these events are related to striking differences between the epididymal cauda milieu, the composition of the ejaculate and the intraluminal fluids of the internal female genital tract to which the cells are sequentially being exposed. The cauda epididymis fluid is acidic in nature [10,11]. The ejaculate is in many species (humans, pigs, horses, among others) and is expelled in fractions, where the first fraction, still acidic due to its dominance in cauda epididymal fluid, holds the emitted vanguard spermatozoa, often involved in fertilization in vivo [12]. The pH of the fluid surrounding ejaculated sperm cells, however, changes dramatically when entering the female genital tract or during in vitro storage in semen extenders or culture media. In vitro storage aims to maintain sperm survival, preserving homeostasis by providing energy and by chelating the calcium present. Upon entry of the female or under conditions of in vitro fertilization (IVF), the sperm cells are, however, confronted with an increase in extracellular pH, either stepwise (in vivo) or immediate (in vitro) [13,14]. As we shall see later, these modifications in extracellular pH (from acidic to basic conditions), as well as exposure to chelates and hormones (such as ovarian progesterone), shall be the determinants of the modifications in the structure, location and function of the CatSper.

The CatSper has been described as being a channel composed of several subunits, all of them building a pore-forming channel [15]. In addition, the CatSper channel has several accessory subunits that contribute to its structure and function [16,17,18,19,20,21,22,23,24]. In 2001, both Ren et al. [16] and Quill et al. [18] described for the first time the main calcium channel in mouse and human sperm cells. Using the novel whole-cell patch clamp technique in human sperm cells, Lishko et al. [9] and Strünker et al. [25] further demonstrated CatSper was the main calcium channel. Of note, the application of the patching technique demands—in order to form a giga-ohm seal—that the patch pipette is placed on the plasma membrane of a cytoplasmic drop since there is no room for patching in other domains of the sperm plasma membrane. While this is understandable from a methodological point of view, it means that the sperm cells used were—per definition—morphologically immature since they retained their cytoplasmic drop. Cauda epididymis sperm cells maintain their distal-migrated drop, which is normally lost after ejaculation. Sperm cells retaining their drop are still definable as immature. Caution is therefore advised when considering these and other studies defining active calcium-conducting channels using the same type of sperm cells [26]. Following these novel publications, the channel was further described using other techniques (immunological) in equines [27] and humans [28,29], as well as being identified in other mammal species, such as ovines [30], porcine [31] and bovines [32]; in most species using ejaculated sperm cells, it is often newly ejaculated but can also be processed by extension in suitable semen extenders. CatSper is not restricted to mammals since it has also been found in sea urchin sperm cells [33,34]. By the same token, it is intriguing that some mammalian species, such as Lagomorpha, have apparently not been studied, perhaps due to difficulties in using suitable markers for the detection of immunolabeling, as most commercial anti-CatSper antibodies are harvested from immunized rabbits.

The reader is invited to consider the many excellent publications reviewing the CatSper ultrastructure as well as the molecular assembly of the channel [26,35,36]. By doing so, the reader might be aware that an overview of the inter-species localization of this conspicuous calcium channel is yet unavailable, particularly considering the differences in the sourcing (epididymal vs. ejaculate, raw vs. processed) of sperm cells.

This review, albeit not exhaustive, describes the inter- and intra-species variations in the localization of the CatSper channel in the sperm plasma membrane, critically considering current shortages in the accuracy of the methods and techniques used. Additionally, it describes the mechanisms of CatSper activation and its role and function during fertilization, with a wider focus on livestock in the hopes of setting paths for future research of the CatSper channel in domestic species. The review is concluded with some considerations about the potential use of CatSper as a diagnostic tool or alternative interventional procedure for sub-fertility in livestock or as a potential target for male contraception in plague species, pets or humans.

## 2. CatSper Structure and Localization

**Figure 1:**
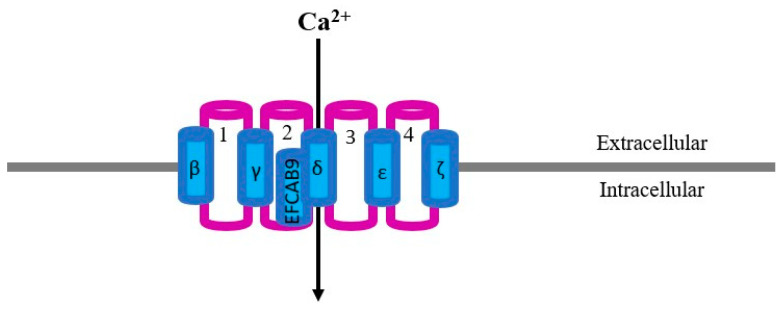
Two-dimensional representation of a simplified model of the CatSper structure: 1, 2, 3 and 4 refer to the main CatSper subunits forming the calcium pore; β, γ, δ, ε, ζ and EFCAB9 refer to the accessory subunits. Protein folding and protein loops have not been considered for this model.

The CatSper structure has been described (Figure 1) as a complex composed of several alpha-subunits (CatSper 1, CatSper 2, CatSper 3 and CatSper 4) building a heterotetrameric pore-forming channel [15,26]. Several accessory units of ancillary transmembrane proteins are further associated with the CatSper pore-forming channel (CatSper β, γ, δ, ε, ζ and EFCAB9) [26], which, together with non-transmembrane cytosolic ancillary-associated proteins [26], all contribute to the structure and function of the channel [16,17,18,19,20,21,22,23,24]. The ultrastructural composition of the channel has been depicted by cryo-electron microscopy [26], immunoelectron microscopy [37] and tomographic image analysis with an overall resolution of 2.9 Å [26,35,36].

Most of our knowledge of the CatSper structure is based on studies in murine epididymal sperm cells. To the best of our knowledge, besides these observations in mice [18,20,38,39,40,41] and humans [28,29], studies of immunolocalization of the four CatSper main subunits in ejaculated sperm cells are restricted to the ovine [30], porcine [31], bovine [32] and equine [27] species. Moreover, sea urchin [33,34] water-expelled sperm cells have been explored. Although the functionality of CatSper has been explored in some domestic animals (Table 1), there is a lack of experimental data regarding the localization and structure of the main and accessory CatSper subunits in other animals, such as rabbits, dogs or wild animals, due to a lack of availability of specific antibodies. These species ought to be studied as soon as the specific antibodies are available. Interestingly, CatSper research in mice has been conducted using epididymal sperm cells, in contrast to all other species, where ejaculated sperm cells have been used. The implication of ejaculation regarding the CatSper structure and function remains, thus, to be elucidated.

**Figure 2:**
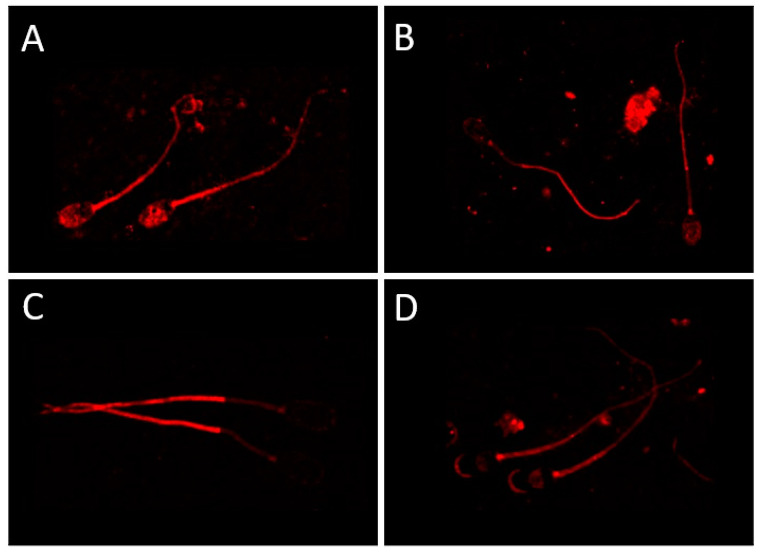
Localization of CatSper in boar sperm cells: (**A**) CatSper 1; (**B**) CatSper 2; (**C**) CatSper 3; (**D**) CatSper 4. Methodology fully described in [31], using commercially available primary antibodies anti-CatSper units (Abcam, Cambridge, UK): CatSper 1 ab101891 (diluted 1:25); CatSper 2 ab101895 (diluted 1:50); CatSper 3 ab101894 (diluted 1:50) and CatSper 4 ab101892 (diluted 1:25). Polyclonal goat anti-rabbit Alexa Fluor 568 (Molecular Probes, Invitrogen, Carlsbad, CA, USA), was used as secondary antibody, diluted 1:1000. The samples were examined under a LSM 700 Zeiss confocal microscope (Carl Zeiss, Sweden) at 630× using DIC and images recorded using ZEN Navigator software (Carl Zeiss, Sweden).

The CatSper channel is apparently located in the principal piece of the sperm tail. Noteworthy, many descriptions allude to the term flagellum, which is incorrect. The sperm tail is morphologically and physiologically different from a flagellum, and proper descriptions of the term and its implications are not always followed, disregarding the different domains of the sperm tail, their construction and function. The experimental results, based on immunocytochemistry, do not fully agree with a conserved localization in the principal piece, given that other locations appear when using ejaculated boar sperm cells (Figure 2A–D) or other species (Table 1) and commercially available antibodies (Figure 2A–D, Table 1) [31]. Therefore, this calls for exploring whether the various locations found relate to the functional activity of the CatSper at specific sperm events. In most species, at least one of the main subunits appears in the plasma membrane of the sperm tail (Table 1).

**Table 1:** Localization, distribution, related function and the methods used to detect the four main subunits of the CatSper.

Receptor	Species	Membrane Domain	Function	Method Used	Reference
CatSper	Mouse	Principal piece	Motility and hyperactivated motility	NB, WB, ICC	Ren et al., 2001 [16]
CatSper 1	Human	Cytoplasmic droplets, midpiece, head	Motility and AR	WB and ICC	Tamburrino et al., 2014, 2015 [28,29]
CatSper 1	Bovine	Principal piece	Hyperactivation	ICC	Johnson et al., 2017 [32]
CatSper 1	Equine	Principal piece	Hyperactivation	PCR, ICC	Loux et al., 2013 [27]
CatSper 1	Sea Urchin	Tail	-	ICC	Loyo-Celis et al., 2021 [34]
CatSper 1	Pig	Acrosome, neck, tail and in cytoplasmic droplets	Motility	WB and ICC	Vicente-Carrillo et al., 2017 [31]
CatSper 1	Ovine	Acrosome and post-acrosome	-	WB and ICC	Vicente-Carrillo et al., 2015 [30]
CatSper 2	Mouse	Tail, principal piece	Motility and hyperactivated motility	WB, ICC	Quill et al., 2001, 2003 [18,38]
CatSper 2	Human	Tail	Motility and male fertility	WB, ICC	Bhilawadikar et al., 2013; Smith et al., 2013 [42,43]
CatSper 2	Pig	Post-acrosome, neck, tail and in cytoplasmic droplets	Motility	WB and ICC	Vicente-Carrillo et al., 2017 [30]
CatSper 2	Ovine	Acrosome and neck	-	WB and ICC	Vicente-Carrillo et al., 2015 [30]
CatSper 3 and 4	Mouse, Human	Acrosome, midpiece, cytoplasmic droplet	AR, hyperactivated motility during capacitation and male fertility	Prediction software, PCR, ICC (only mouse)	Lobley et al. 2003; Jin et al., 2005, 2007 [20,39,40]
CatSper 3	Pig	Neck, tail and in cytoplasmic droplets	Motility	WB and ICC	Vicente-Carrillo et al., 2017 [31]
CatSper 3	Ovine	Post-acrosome and principal piece	-	WB and ICC	Vicente-Carrillo et al., 2015 [30]
CatSper 3	Sea Urchin	Principal piece	Chemotaxis and sperm motility	ICC	Seifert et al., 2015 [33]
CatSper 4	Mouse	Principal piece	Hyperactivated motility and male fertility	ICC	Qi et al., 2007 [41]
CatSper 4	Pig	Post-acrosome, neck, tail and in cytoplasmic droplets	Motility	WB and ICC	Vicente-Carrillo et al., 2017 [31]
CatSper 4	Ovine	Post-acrosome	-	WB and ICC	Vicente-Carrillo et al., 2015 [30]

NB: Northern blot; WB: Western blot; ICC: immunocytochemistry; PCR: polymerase chain reaction.

However, the localization of each main subunit is not always determined by immunolocalization, as most of the literature—beyond describing the immunolabeling of one of the subunits—assumes the other ones are there by using predicting software (see Table 1 and references therein). Deeper research is therefore required, not only on the locations of all the main and accessory subunits of the CatSper via immunolocalization but also considering whether the location mirrors functional events.

## 3. CatSper Activation

Activation of the CatSper channel implies the entrance of calcium in sperm cells in relation to other events, such as their exposure to progesterone and/or alkalinization [9,31], two factors related to sperm entry on the internal female tract, as described above. The use of intracellular calcium deposits implies their proximity to the sperm neck, and the rapid response that the CatSper conveys is most likely related to its localization in the midpiece and principal tail domains, involved in the homeostasis of sperm motility before and after sperm capacitation has occurred. Such a location has been registered in virtually all species explored when considering ejaculated sperm cells (Table 1).

Progesterone activates the CatSper channels, inducing both increased and serial oscillations in intracellular calcium concentrations, as depicted using a specific blocker (RU1968) or an SKF inhibitor in human sperm cells [44]. CatSper activity in human sperm cells is apparently independent of the cAMP/PKA-signaling pathway [45], as described using CATSPER2 gene deletion in deafness-infertility syndrome (DIS). Interestingly, apart from human sperm cells, there are no studies of whole-cell patch clamping in any other species. This is not surprising since the prerequisite for the application of patching is the presence of a cytoplasmic droplet, demanding the use of either epididymal sperm cells or immature, ejaculated sperm cells. As described earlier, the cytoplasmic droplet (which migrates from a proximal to a distal position in the midpiece of the sperm tail) is normally lost at ejaculation [46]. When ejaculation occurs without resting periods, the number of sperm cells retaining cytoplasmic droplets increases, a sign of incomplete sperm maturation in the epididymis, as evidenced in breeding sires [1,11,46].

In morphologically normal ejaculated boar sperm cells, the response to progesterone can be affected using inhibitors of ABHD2 and of CatSper [47], suggesting that progesterone could also act on CatSper in ejaculated sperm cells from other species [48]. Additionally, cortisol can inhibit the progesterone-mediated activation of the channel in human sperm cells [49]. Calmodulin can reduce the expression of CatSper 1 [50] in mouse epididymal sperm cells; however, this has not been explored in ejaculated sperm cells or other species.

The sperm intracellular calcium concentration is regulated by the balance between a calcium influx via membrane channels, such as CatSper, and the storage of calcium in intracellular deposits [51]. CatSper activation is also dependent on intracellular tyrosine phosphorylation signaling, which regulates the intracellular pH by controlling the intracellular calcium concentration [52,53]. Fine-tuned calcium homeostasis guarantees the balance in sperm reactivity; therefore, sperm cells are ready to overcome the pre-fertilization and fertilization events on time (see the next section). Although CatSper activation implies the entrance of calcium, CatSper genes can also be up or downregulated in sperm cells. In boar, the *Prunuis japonica* extract enhances sperm motility [54], as analyzed after the use of the inhibitor, Mibefradil. Another extract, *Putranjiva roxburghii*, upregulated a series of genes in bovine sperm cells, including CatSper [55]. Epicathenin prevents cryocapacitation-like events related to the loss of the CatSper isoforms 1 and 2 [56], while *Fumaria parviflora* enhances the mRNA levels of CatSper 1 and 2 in rat epididymal sperm cells [57]. On the other hand, CatSper 1 and 2 are downregulated in heat-stressed rat sperm cells, perhaps in relation to the well-recognized impairment of spermatogenesis by increased temperature [58]. Interestingly, Tmem249 uniquely uses a CTG start codon to encode CATSPERθ, the latter stabilizing the CATSPER4 subunit and its conformational ordering [59].

In boar sperm cells, previous studies at the mRNA level confirm that bicarbonate triggers changes in the expression of the CatSper-γ subunit mRNA transcript, as well as the transient receptor potential cation (TRPC) channels 3, 4, 6 and 7 [60]. In addition, Taxifolin increased the protein levels of CatSper in duroc boars [61]. Calcium is not an exclusive molecule in CatSper activation since there are more receptors, such as TRPCs, also affected by K^+^ and Na^+^ [62]. In mammals, up to seven TRPC genes have been identified, and they are related to the phospholipase C (PLC)-dependent calcium influx [63]. The TRPC presence and distribution in sperm cells vary across mammalian species [64]. Variation during the in vitro capacitation of boar sperm cells has been described [60], highlighting the necessity of carrying out new mechanistic experimental setups, including not only CatSper but also other calcium-dependent channels.

Information in the literature is undoubtedly still unavailable regarding the activation and molecular mechanisms of CatSper in the sperm cells of several domestic species, such as rabbits and dogs, as well as in wild animals. Likewise, comparisons in sperm cells derived from various sources and handling are yet missing. This information shall be relevant for deepening the understanding of animal reproduction as well as increasing our knowledge of the species used as research models.

## 4. Role of CatSper in Fertilization

After ejaculation, sperm cells are unable to fertilize an oocyte either in vivo or in vitro without undergoing sperm capacitation, hyperactivation of sperm motility and the acrosome reaction [4,13,65,66]. All of these processes are calcium-dependent since an increase in intracellular calcium concentration will trigger them [51]. Surrounded by an acidic environment, as is the case in the epididymal cauda [67], the CatSper is mostly closed, regardless of the intracellular Ca^2+^ concentration, which might indicate proper homeostasis and the stability of the plasma membrane, a so-called “resting state” [26]. When alkalinity increases (as in the oviduct, [46]), the CatSper apparently opens, denoting a high sensitivity of the channel [26]. Such increased sensitivity might be further potentiated by an influx via the calcium channels [51,68] of extracellular Ca^2+^ and by Ca^2+^ mobilization from intracellular deposits, such as the endoplasmic reticulum and mitochondria [69]. The first source appears more relevant and might explain why Ca^2+^-chelators (as EDTA) are needed in semen extenders to keep the CatSper closed during the period when sperm cells are stored in vitro [31]. The CatSper channel seems essential for sperm motility and male fertility, as CatSper-null sperm cells are unable to hyperactivate and fertilize an oocyte [2,16,23,39].

Sperm capacitation, first evidenced by Austin and Chang in 1951 [70,71], is a series of processes that ejaculated sperm cells must overcome in the female genital tract that allow them to gain their fertilizing ability [13]. Among others, sperm capacitation mainly involves the destabilization and reorganization of the sperm plasma membrane, as well as increases in intracellular calcium, intracellular alkalinization and increases in protein-tyrosine phosphorylation [7,72,73,74,75]. Increases in intracellular calcium are mediated by several calcium channels, with the main calcium channel of sperm cells (CatSper) taking a central role [9,31,32,34] (Table 1). As demonstrated in pig sperm cells, CatSper is activated during sperm capacitation [31] (Table 1) and is reflected in its immunolocalization at specific plasma membrane domains, as commented above. Surprisingly, complementary studies about the role and implication of CatSper during capacitation are yet to be conducted in other species.

Once sperm cells have completed capacitation, advancement along the oviduct on their way to the oocyte requires they become free from the storage tubal sites and advance in the viscous oviductal fluid [13] by displaying a motility pattern called “hyperactivated motility”, characterized by a strong wipe-like beating of the sperm tail [76]. Hyperactivation of sperm motility is calcium-dependent and clearly linked to CatSper [2,38,41,68] (Table 1). A complete CatSper channel, i.e., with all subunits, is required for hyperactivated motility and, ultimately, for male fertility [41]. Sperm cells from CatSper-null animals are infertile, most likely because they cannot properly capacitate/hyperactivate [26]. CatSper has a clear role in sperm hyperactivation via the EFCAB9 protein, a subunit of the CatSper channel with pH-sensing and Ca^2+^-binding actions, as EFCAB9-null sperm cells are unable to hyperactivate and thus fertilize the oocyte [26,77].

Finally, prior to fertilization, sperm cells undergo exocytosis of the acrosome (which implies disruption of the covering apical sperm head plasma membrane domain and the subjacent outer acrosome membrane) in the so-called “acrosome reaction” [72]. A true, physiological acrosome reaction can only take place after sperm capacitation (and the parallel hyperactivation of the motility) when the ZP is contacted and thereafter penetrated to allow for the fusion of the equatorial plasma membrane domain of the sperm cell and the oocyte oolemma [72]. Spontaneous exocytosis of the acrosome can occur during cell death or during pharmacological/experimental modification of the sperm surroundings, i.e., using Ca^2+^ ionophores in the absence of the oocyte or of ZP proteins [66]. The process of acrosome reaction is also calcium-dependent and thus directly related to CatSper, although there are not many recent studies regarding CatSper and the acrosome reaction performed beyond mouse and human sperm cells [20,28,29,39,40] (Table 1). The Ca^2+^ and pH sensitivity displayed by the CatSper during its activation could suggest a well-conserved mechanism for sperm pre-fertilization events without implying substantial changes to its immunolocalization, probably since the membrane domains where the CatSper is displayed are maintained throughout capacitation and the acrosome reaction. Being a calcium channel and with all the pre-fertilization and fertilization processes being calcium-dependent, the CatSper seems to be directly implicated in male fertility [78,79,80], as CatSper-null males are infertile [39]. It would be interesting to explore the function, mechanisms of action, and the ultimate role of CatSper in relation to the reproductive cycle, as it has been proven that other sperm membrane receptors display variations according to seasonality [81]. In addition, it would be interesting to investigate the function of this calcium channel in species where reproduction follows an induced-ovulation pattern, such as cats, rabbits or camels. Up to now, and to the best of our knowledge, no studies about seasonality or reproductive cycle interactions have been conducted in relation to the CatSper channel.

## 5. Mechanisms of Sperm Interaction with the Surroundings and Their Relationship to CatSper

Sperm cells are highly interactive cells that interact both in vivo and in vitro with a high variety of media, fluids and cells [3]. Once sperm cells are produced in the testis, they mature in the epididymis and wait in its caudal segment until ejaculation occurs [1,11]. In the cauda epididymis, the low pH of the slightly hyper-osmotic intraluminal fluid maintains the sperm cells quiescent [10,67]. After ejaculation, the seminal plasma increases the surrounding pH and levels of osmosis, activating the ATP-dependent sperm motility [82,83]. Calcium entry to sperm cells and the mobilization of intracellular calcium deposits regulate sperm homeostasis [51,68,84]. In order to survive, sperm cells count on a series of membrane receptors that help overcome the constantly changing environment: from the cauda epididymis, the ejaculate and—in vivo—intraluminal fluids of the female (cervix, uterus, oviduct), or when in vitro, of different buffer media, e.g., for in vitro fertilization [85].

Both in vivo and in vitro, sperm cells must handle osmotic variations to adapt to changing environments [13]. Osmotic adaptation is regulated via aquaporins (AQPs; [86]). When in vivo, contact with the seminal plasma and the female’s genital tract fluids stimulates the activation of these water channels, while, in vitro, AQPs help adapt to sperm handling using semen extenders, refrigeration and freezing/thawing [86].

Contact with the seminal plasma implies sperm cells interact with a plethora of compounds, such as glucose, fructose, bicarbonate, calcium, zinc, proteins, etc. [12], including seminal plasma extracellular vesicles (SEVs) [87]. Sperm–seminal plasma interactions cause both positive and negative circumstances for sperm survival; when in vivo, nutrition, vehicle and protection are provided [88,89], while when in vitro, sperm cells are better preserved with a lower ratio of seminal plasma:extender [90,91]. In vivo, SEVs enrich sperm cells with exogenous essential regulatory molecules (RNA, etc.) [92,93,94]. The SEVs are crucial extracellular structures that interact with sperm cells and even provide the latter with their membrane after SEV-sperm fusing, thus incorporating into the sperm cells a plethora of channels and receptors with key roles in sperm function [92,94,95,96,97,98]. Further implications of SEVs in sperm function and membrane exchanges are expected to be elucidated by future research.

**Figure 3:**
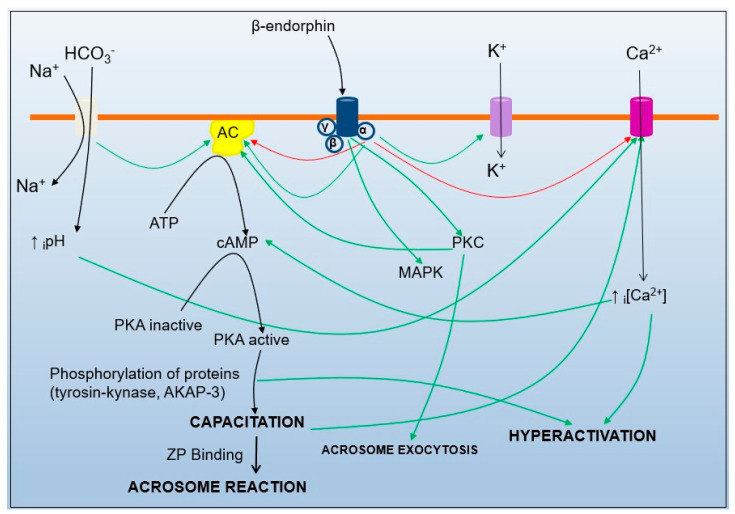
Intracellular pathways and sperm outcome after intracellular alkalinization due to bicarbonate entrance in sperm cells (gray), activation of CatSper channel (pink), the opioid receptors (blue) and potassium channels (purple). Green lines indicate stimulation or activation, and red lines blockade or inhibition. Modified from [3].

The fine regulation of sperm motility and the acrosome reaction is calcium-dependent; however, calcium is not the only molecule implicated in this complex process (Figure 3). Other membrane calcium channels and receptors, as well as ion channels for potassium, sodium or chloride, and receptors for opioids, progesterone, hyaluronic acid, etc., all interact, preserving sperm homeostasis and modulating functional changes [3,85]. The proper temporal activation/blockade of each of the sperm membrane receptors and channels and the balance of them would contribute to fulfilling the final fate of the sperm cells: to fertilize the oocyte [3].

Intracellular pathways triggered after CatSper activation are shared pathways with many other intracellular signaling routes, for instance, when CatSper is mainly activated by intracellular alkalinization and progesterone (see above), which indeed will lead to calcium entry to sperm cells [9]. When calcium and bicarbonate enter, soluble adenylyl cyclase is activated, and this initiates the PKA pathway and the phosphorylation of proteins (such as tyrosine kinase and AKAP-3 [37,99,100]), also causing changes in the motility pattern towards hyperactivation. In addition, when sperm cells follow their path towards the oocyte, they are exposed to increasing concentrations of endorphins and enkephalins (with the highest concentrations in the pre-ovulatory follicular fluid [101]). These endogenous opioids bind, with their affinity depending on the subtype [102], to G-protein-coupled opioid receptors. The signal transduction of the opioid receptors blocks the calcium channels [103] and opens K^+^ channels, causing depolarization of the sperm membrane and decreasing motility.

Moreover, low opioid doses or the short-time stimulation of opioid receptors can block soluble adenylyl cyclase [104], causing inhibition of sperm motility because of a lack of production of cAMP because of the blockade of the PKA pathway. On the other hand, higher opioid doses or longer stimulation (implying sustained stimulation) of the opioid receptors cause an opposite effect, stimulating soluble adenylyl cyclase [104], which stimulates the PKA pathway by increasing the production of cAMP and sperm motility, ultimately leading to acrosomal exocytosis (Figure 3). However, this mechanism can end in acrosomal exocytosis and cell death if sperm cells have not reached the ZP and thus are unable to fertilize the oocyte. This biphasic effect of the opioid receptors [105], because of acute/chronic stimulation, has also been demonstrated by us in boar sperm cells when challenged in vitro with the µ-opioid agonist morphine [106].

In summary, with calcium being essential for the regulation and activation of all pre-fertilization and fertilization processes, it remains for us to study how sperm cells can adapt to these events and still preserve fertilization potential, assuming the sperm cell is a terminal cell, specialized in interacting with other cells (the epithelial lining or the oocyte vestments) or cell products (as secretion or the ZP). Calcium and its main mobilizing membrane channel, CatSper, are extraordinarily relevant for sperm function [39]. Keeping in mind that sperm adaptation, homeostasis and function depend on several mechanisms, it is worth noting that calcium—transported through the CatSper—is central to all these mechanisms and, thus, possibly the most relevant ion for sperm cells. Without CatSper, the calcium influx in sperm cells would be minimal, and this would render sperm cells unable to fertilize an oocyte [38,43]. Because of the latter, CatSper appears as a very interesting target for diagnosing idiopathic male-related infertility as well as being a potential target for male nonhormonal contraception [100].

## 6. CatSper, a Potential Target for Male Fertility Intervention?

The development of male contraception therapy has been a field of interest in the pharmaceutical industry for a very long time [107]. In this context, the use of CatSper as a target has been considered as an alternative to explore [100].

It has been shown that CatSper is exclusively expressed in the testes [20,40] and that this has a crucial role in male fertility [39]. These two circumstances make CatSper a possible ideal target for nonhormonal male contraception [108], aiming to modulate, among other functions, sperm motility. This approach has been extensively used for high-throughput drug screening [109]. Moreover, knock-out mice for CatSper are infertile [38,43], which proves once again the relevance of CatSper in male (in)fertility.

The idea of using the CatSper as a target for contraception has been periodically mentioned in the literature during the last 20 years [100]. Yet, no contraceptive method based on the inhibition of CatSper has become available for male contraception; however, the related ethical concerns have not been discussed deeply enough. One possible reason for the lack of such a contraceptive method is that there is no specific inhibitor for CatSper currently available. Most of the inhibitors that are used in functional studies of CatSper are calcium channel inhibitors (such as Mibefradil or NNC 55-0396, [28,31]). With CatSper being the main calcium channel in sperm cells, a general inhibition of calcium channels will mainly inhibit CatSper [31]; however, we cannot warrant that the inhibition of other calcium channels would disrupt the calcium homeostasis in other cells of the organism. Such a scenario is serious and, therefore, ethically complicated. An unspecific calcium channel inhibitor cannot be used as contraceptive therapy, as it will have enormous undesired side effects. Up to now, the only way of specifically blocking the CatSper channel is via a knock-out [110], which can obviously not be completed, either in humans or in adult individuals of other species. The development of specific CatSper blockers has nevertheless lately started [111,112], which opens the door for deeper and careful research in the area, considering the substantial ethical concerns and long-lasting effects their use implies. Obviously, inhibition of CatSper to fight fertility in invasive species, wild rodents (such as rats in a metropolis, where numbers are extremely high) or stray dogs to provide long-lasting control of animal populations without the need for extermination using toxic substances, etc., might be tempting to study. This approach is, however, neither free from ethical nor of potential long-lasting undesirable effects.

But how about more specifically identifying sub-fertile livestock? Breeding sires have been used to deliver semen for artificial insemination (AI) for 100 years, with some species, such as dairy cattle or porcine, being AI-bred to 100%. Despite the selection of these sires for sperm quality and fertility after AI, the current methods of semen evaluation can still not detect sub-fertile males before AI of sufficient numbers of females has been evaluated, implying costly losses [46]. Data from mice studies suggest there is a threshold for intact CatSper to define a fertile spermatozoon by the order of 30% [37]. To use mapping of CatSper as a biomarker in livestock might be difficult or costly; however, considering the refractoriness towards progesterone stimulation that has been recorded, the screening of sub-fertile males for CatSper activation potential might be an option to gather whether there is a threshold for a minimal CatSper function. Whether this threshold can be manipulated will depend on our deeper knowledge of how gating works for the CatSper when changing from an inactive form to an activated one. The gains obtained in identifying the molecular structure of the CatSper [26] will undoubtedly contribute to the development of structure-based compounds that could modify (i.e., enhance or inhibit) the function of the CatSper. Without this basic knowledge, procurement of therapeutic gains is probably utopic; however, the design of biosensors for diagnostic aims will probably be reached sooner.

In summary, the potential use of the CatSper channel as a target for intervention regarding fertility modification, e.g., contraception in plague animals, pets, wild/feral animals or humans, or the improvement of fertility in sub-fertile individuals, is so far to be considered if not utopic, at least far reached. The ethical concerns to which such interventions are attached shall probably be so large that such an endeavor is not feasible. However, the use of CatSper as a biosensor for fertility in species of productive interest or of main diagnostic value for sub-fertile patients is more attractive. The latter are areas where we should expect deeper, careful research, thus completing the data we are still missing for this conspicuous calcium channel.

## 7. Concluding Remarks

The CatSper channel seems to be, as the experimental data show, a relevant channel for sperm cells with a clear implication for sperm function. Many studies performed in the last two decades have deepened our understanding of sperm physiology. Although the CatSper gene sequence seems to be conserved among several species [113], there are studies denoting the eventual biological variations in location and reactivity (see Section 2 and Table 1), calling for deep comparative studies about its structure and function.

Interestingly, immunocytochemistry (ICC) studies, when using polyclonal antibodies and immunofluorescence (either using an epifluorescence or a confocal microscope) in different species, showed that CatSper subunits are seemingly not located in the same domain of the sperm membrane (Table 1). This point highlights a relevant issue: Is the CatSper really formed by several independent subunits? All the functional studies (activation/blocking of the channel) have been completed with the whole CatSper, not putting into relevance if one subunit is being blocked or activated. Is each subunit able to work independently? Are our proteomic or our antibody-detection analyses wrong?

The structure of the CatSper channel is usually defined by a proteomic approach and then confirmed by ICC [20,29,31,38,39,40]. Lately, the fine structure of the CatSper has been revealed by transmission electron microscopy (TEM [26,35,36]). Yet, these TEM studies are only based upon one of the CatSper subunits (CatSper 1, [26,35,36]), resulting in the assumption that the entire CatSper channel, at least for murine sperm cells, is located in the principal piece of the sperm tail (erroneously defined as the “flagellum” [26]), which is, apparently, not the case for other species [29,30,31] where the midpiece is also involved (Figure 2A–D). Interestingly, the ICC shows different results in terms of the location of the CatSper channel in different species and, within a particular species, the different subunits are not even located in the same membrane domain (Figure 2A–D) [31].

There might be, of course, inter-species differences in terms of the location of CatSper, as it happens in terms of the mechanisms of activation [9,31]. However, according to the proteomic studies on the CatSper structure, all CatSper subunits shall work together to build a pore-forming channel. Thus, no differences in the membrane domain should appear for the CatSper subunits, and we expect to find all of them together. 

Based on our previous studies in sperm membrane protein determinations [86], the most plausible explanation is that there is a consistent lack of specificity in the antibodies used, considering several authors working with different antibodies reached different conclusions (cited in [86]). This issue should make the scientific community reflect on the validity and reliability of the obtained results, specifically when using polyclonal antibodies. As technology development moves forward, new microscopes with higher resolution appear, better software is available, and scientists are better trained. However, all this development is worthless if our primary system of detection is not specific enough, and thus, there are doubts regarding what is exactly detected.

Considering the latter, deeper studies regarding the immunolocalization of all the CatSper subunits for each species shall be conducted, given that detecting only one subunit and assuming all the others are there is not enough proof. Finally, there are still many species where no studies have been performed about the presence, structure, function or relation between seasonality and the reproductive cycle of the CatSper. In addition, most functional studies of CatSper, up to date, have used the generic calcium channel inhibitors Milbefradil and NNC55-0396 (see references in Table 1), while new and specific CatSper blockers have been developed in the last years [109,112].

This scenario settles the path for further and deeper research into the main calcium channel, CatSper, particularly to explain if a variation in location among species might simply be related to the differences in the identification laboratory methods used or if they even imply they do not have any biological function. This is relevant, given a generally constant location among species would make the CatSper a target molecule for intended modifications of function, attempting a diagnosis of idiopathic sub-fertility or the highly controversial, unethical interventional manipulation of male contraception.

## Data Availability

Not applicable.
